# Hepatotoxicity Induced by “the 3Ks”: Kava, Kratom and Khat

**DOI:** 10.3390/ijms17040580

**Published:** 2016-04-16

**Authors:** Flaminia Pantano, Roberta Tittarelli, Giulio Mannocchi, Simona Zaami, Serafino Ricci, Raffaele Giorgetti, Daniela Terranova, Francesco P. Busardò, Enrico Marinelli

**Affiliations:** 1 Department of Anatomical, Histological, Forensic and Orthopedic Sciences, Sapienza University of Rome, 00161 Rome, Italy; flaminia.pantano@uniroma1.it (F.P.); roberta.tittarelli@uniroma1.it (R.T.); giulio.mannocchi@uniroma1.it (G.M.); simona.zaami@uniroma1.it (S.Z.); serafino.ricci@uniroma1.it (S.R.); daniela.terranova@uniroma1.it (D.T.); enrico.marinelli@uniroma1.it (E.M.); 2 Section of Legal Medicine, Università Politecnica delle Marche, 60121 Ancona, Italy; r.giorgetti@univpm.it

**Keywords:** kava, khat, kratom, hepatotoxicity, herbals, herb induced liver injury

## Abstract

The 3Ks (kava, kratom and khat) are herbals that can potentially induce liver injuries. On the one hand, growing controversial data have been reported about the hepatotoxicity of kratom, while, on the other hand, even though kava and khat hepatotoxicity has been investigated, the hepatotoxic effects are still not clear. Chronic recreational use of kratom has been associated with rare instances of acute liver injury. Several studies and case reports have suggested that khat is hepatotoxic, leading to deranged liver enzymes and also histopathological evidence of acute hepatocellular degeneration. Numerous reports of severe hepatotoxicity potentially induced by kava have also been highlighted, both in the USA and Europe. The aim of this review is to focus on the different patterns and the mechanisms of hepatotoxicity induced by “the 3Ks”, while trying to clarify the numerous aspects that still need to be addressed.

## 1. Introduction

Liver damage caused by herbal medicines, also called herb induced liver injury (HILI), is a rare event that occurs in a small number of susceptible individuals [1,2,3]. Its characteristics are similar to those of drug induced liver injury (DILI). Commonly in HILI and DILI cases, injury is related to idiosyncratic reaction that occurs at recommended doses [4]. The diagnosis of HILI generates both regulatory and clinical challenges. The assessment of causality according to Teschke *et al.* [2] should be performed using the Council for International Organizations of Medical Sciences Scale (CIOMS), also known as RUCAM (Roussel Uclaf Causality Assessment Method) [4], which is specific for the liver and validated for hepatotoxicity. Moreover, even though herbal supplements are under regulation in many countries, standard levels could dramatically differ, including the possibility of herb misidentification, the presence of impurities and/or adulterants. Naranjo scale, the *ad hoc* approach and the World Health Organization (WHO) method are not equal to the CIOMS scale in the evaluation of HILI causality. However, if HILI is caused by herb intake, caution is mandatory and the compound must be avoided, even when lacking causality assessment. Moreover, in order to achieve a diagnosis, it is necessary to exclude several intra- and extra-hepatic causes. The most suitable criterion for HILI causality assessment has been defined by some authors [5] to be a positive re-exposure reaction, if evaluated by established test criteria.

### 1.1. Kava: General Concepts

**Figure 1:**
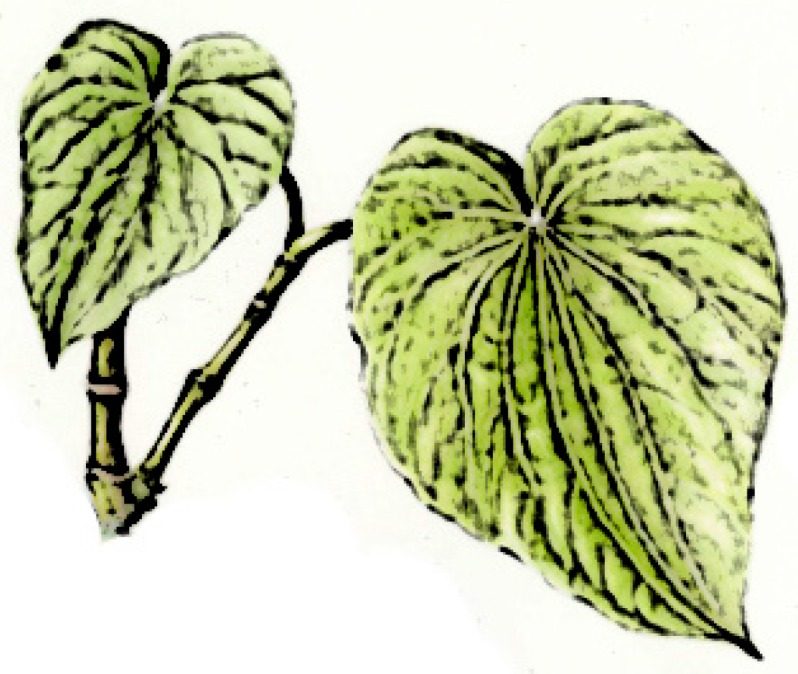
Kava leaves.

Kava is a traditional Pacific beverage made from the roots and stems of *Piper methysticum* Forst. f. (“*awa*” meaning bitter in Hawaiian) plant, which belongs to the pepper family [6,7,8] (Figure 1). In 2002, the Vanuatu government approved the Kava Act [9], in which kava is identified in different cultivars and categorized: noble cultivars (long history of safe use for traditional purposes), medicinal (ancient and confirmed history of useful properties amongst traditional herbalists for therapeutic properties), two days cultivars (able to intoxicate for two days and banned from export) and *wichmannii* cultivars (*Piper wichmannii*, ancestor of *P. methysticum*, wild variety banned from export). One of the favored noble cultivars is Borogu, which contains high quantities of kavain and dihydrokavain, and is known for its quick effects [10]. The drinking of kava was sacred and its use was limited to defined social classes such as priests and chiefs. The traditional drink is known to decrease anxiety and fatigue, it produces a sociable attitude, relieves pain and induces sleep [11]. It was consumed for its intoxicating properties in countries where alcohol fermentation only arrived at a later date. This aqueous compound has been drunk for centuries in the southern Pacific, without any apparent consequences, apart from kava dermopathy and if taken heavily it is associated with a raise of liver enzymes, primarily γ-glutamyl-transpeptidase (γ-GT) even causing sudden cardiac death [12,13,14,15,16,17,18]. However, in Western countries, kava extracts (obtained by acetonic or ethanolic extraction) have been used mainly for the treatment of anxiety [19] disorders and restlessness and have been linked with hepatotoxicity, especially in females [20], with a mean average of 111 days from the first exposure until the onset of liver impairment. Other authors suggest that kava hepatotoxicity can also be observed after the intake of traditional aqueous kava preparation and therefore it should not be linked to acetonic or ethanolic extraction, suggesting that the toxicity is due to the kava plant itself or to the low quality of kava cultivar used and, more generally, to a lack of standardization in the production of kava supplements or contamination [21,22,23,24]. As suggested by Lebot [25], major criteria for kava quality are: chemotype, total kavalactone content, preparation and dilution method, part of the plant used, and dry or fresh material use. According to Sorrentino *et al.* [26], kava products are to be considered safe at the dosage of 100 mg/kg/day (even though this dosage corresponds to 10 times the recommended daily dose) and, according to pre-clinical, clinical and toxicological studies, do not cause toxicity [27]. The first cases of kava related hepatotoxicity were described by Strahl *et al.* [28] in 1998; since then, more than 100 cases of severe liver injury following kava exposure have been suspected worldwide. Kava products have been withdrawn from Europe, USA and Australia; moreover, Food and Drug Administration (FDA) and Center for Disease Control (CDC) warnings and European regulatory agency warnings have been diffused since 2002 [29,30]. In Italy, kava products were also withdrawn from the market in 2002 [31]. However, possible damage mechanisms have not yet been fully established, probably because of the numerous variables involved in kava products and as the kava plant itself contains more than 40 constituents.

### 1.2. Kratom: General Concepts

**Figure 2:**
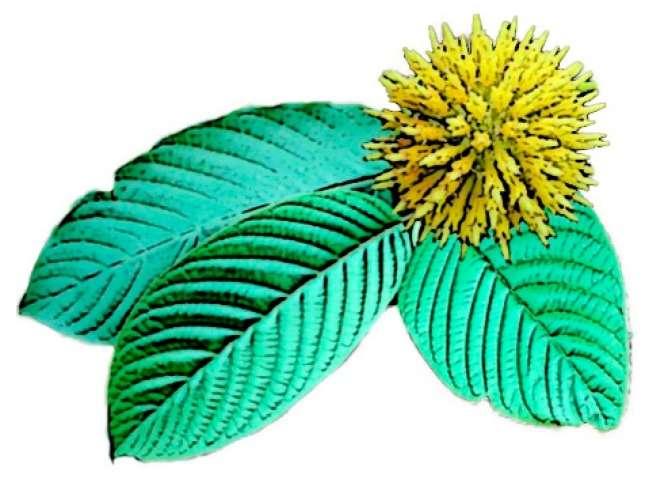
Leaves and flower of *Mitragyna speciosa*.

Kratom is a natural psychoactive preparation obtained from a plant known as *Mitragyna speciosa* Korth, belonging to Rubiaceae or coffee family [32]. The tree of this genus has a widespread distribution, both in the tropical and subtropical regions of Southeast Asia and in several areas of Africa. It is also known as “*biak-biak*” or *Ketum* in Malaysia; *Kratom*, *Kakuam*, *Kraton*, *Ithang* or *Thom* in Thailand [33,34,35,36]; and *Mambog* in the Philippines [37]. The tree has wide, shiny, oval-shaped bright green-colored leaves that can grow up to 18 cm in length and 10 cm in width. The flowers of the plant are yellow and have a spherical shape with a cluster organization [37,38] (Figure 2).

The leaves of *M. speciosa* (*Mitragyna speciosa*) can be chewed fresh or dried, smoked or infused like an herbal tea with sugar or honey to reduce its typical bitter taste [32]. The natives of the Southeast Asian regions consumed the leaves and the smaller stems, both for their coca-like stimulant ability [39]; when consumed at low doses (approximately 1–5 g of fresh leaves), to combat fatigue and to improve working endurance under the blazing sun; and as an opium substitute [40], for their morphine-like effect at high doses (approximately 5–15 g of fresh material) [37], in cases of opium addiction [41] and for the self-treatment of withdrawal symptoms. If taken at very high doses (>15 g), kratom causes the onset of a sedating status with hypnotic effects and stupor experiences, similar to those caused by opioid consumption [42]. The first reports of kratom use for the treatment of opium addiction date back to 1836, as reported by Burkill [43], and the method of preparation for its consumption was first described by Wray in 1907 [44]. Kratom preparations are also taken in Thailand and Malaysia for medical use in cases of chronic musculoskeletal pain, enteritis, worm infections [45], cough, diabetes and hypertension [32]. Benefits reported are also connected to its analgesic, anti-inflammatory and antipyretic effects. Currently, the use of kratom for recreational purposes has widely spread in Europe and in the US because it is easy to purchase on the Internet [46] and also because it is not a controlled drug, making it a “legal high” in many countries [32]. *M. speciosa*, as reported by several authors, can also be used as a component of herbal preparations, as in the case of “Krypton”, a mixture containing powdered kratom, caffeine and *O*-desmethyltramadol [47], which caused the death of nine subjects in Sweden in a short period of time [48,49]. Recently, in the southernmost provinces of Thailand, the consumption of a cocktail called “4 × 100”, containing three basic components: *M. speciosa* leaves, caffeine (from cola drink), codeine (an antitussive) or diphenhydramine (an antihistamine), to which is added a fourth ingredient chosen among antidepressants, anxiolytics or analgesics (paracetamol or tramadol) agents, has caused several deaths among young Muslims, who drink it for its effects similar to those produced by drinking alcohol, which is forbidden in their religion [50]. Because of its ability to produce alterations to the state of consciousness, mitragynine, the main alkaloid compound present in kratom, and its derivative 7-hydroxymitragynine (7-HMG), are controlled drugs in several countries, such as Denmark, Lithuania, Latvia, Romania, Poland, Sweden *etc.* [51]. Furthermore, kratom is illegal in Australia, Malaysia, Burma and Thailand, while in New Zealand *M. speciosa* and mitragynine, are included in the Medicines Amendment Regulations Act. South Korea, Germany and Israel have also enacted new regulations in controlling kratom and its constituents [37,52]. Instead, in the US, kratom is not scheduled under the Controlled Substances Act, but in 2010, the Drug Enforcement Administration (DEA) included it in the Drugs and Chemicals of Concern list, because kratom is not considered a legitimate remedy for any medical purpose [32,37,42]. Although kratom seems to be safe when administered at 1–10 mg/kg doses (which represents a sub-chronic dose), after prolonged exposure to a 100 mg/kg dose, as demonstrated in the experiments on Sprague-Dawley rats conducted by Sabetghadam *et al.* [53], it causes biochemical and hematological changes with histopathological alterations in several tissues (liver, kidney and brain). In the same paper, the authors reported that kratom users consume about 67.5–75 mg of kratom per day and that no adverse effects were shown while only after prolonged exposure to a higher dose of kratom, clinical signs of toxicity were highlighted [41]. Only a few papers [54] report liver damages or hepatotoxic sequelae related to kratom use, and also in these cases, the authors highlighted the difficulties of a correlation between kratom consumption and hepatic injuries, which was more likely to be associated with the extraction process of the alkaloids or to the presence of contaminants in the herbal products [52]. Moreover, causality has not yet been accurately established for kratom, as CIOMS scale (RUCAM) has not been applied to suspected cases. Further information is available in the drug user web fora [55]; although these are not official sources, they could represent an incentive for researchers to enhance their knowledge of its metabolism and its adverse effects in humans.

### 1.3. Khat: General Concepts

**Figure 3:**
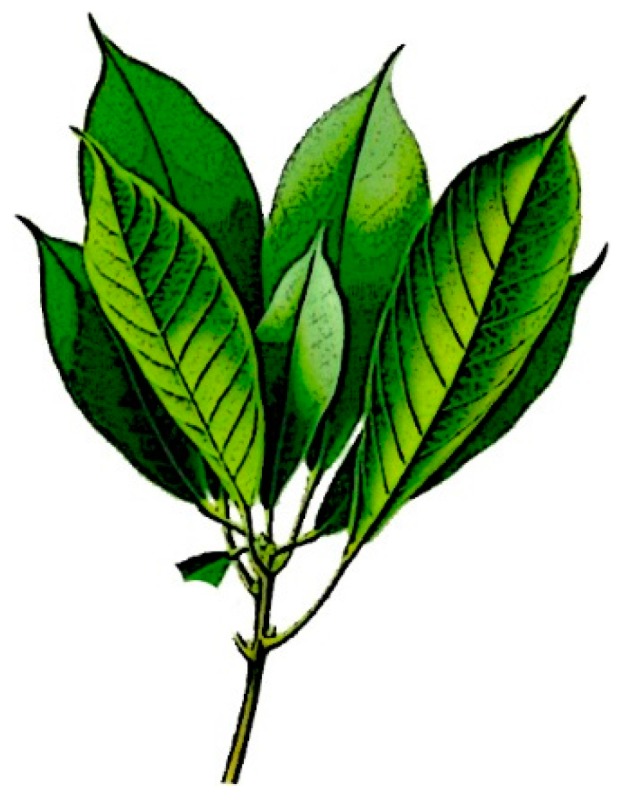
Khat leaves.

*Catha edulis* (khat) is a plant (Figure 3) with psychoactive effects, the most widely used in the world. Grown in the east of Africa, in the Horn of Africa and southwest of the Arabian Peninsula, the foliage and branches are used daily by more than 20 million people [56,57]. Historical evidence highlights that khat has been in use since the 13th century in Ethiopia and in the east of the Arabian region, before the cultivation of coffee. In the last thirty years it has become available and its use has extended in the world, including North America and Europe. The main consumers in the West are groups of immigrants from eastern Africa and the Middle East [56], especially immigrants from Somalia, Ethiopia and Yemen [58]**.** According to the World Health Organization, khat is a drug of abuse, due to the health problems it can cause. Although it is still legal in many countries, it is forbidden in almost all Western countries such as Germany, France, Netherlands, United States and Canada, while it is allowed in Somalia, Djibouti, Ethiopia, Yemen and Israel [56]. *Catha edulis* contains a number of substances, among which the most important, cathinone and cathine, have a similar effect to amphetamine. When chewing khat, cathinone is released producing a feeling of euphoria. The production of cathine and norephedrine has been detected after cathinone is metabolized, and its structure is related to amphetamine and adrenaline (epinephrine) [58,59]. Cathinone also reduces appetite through an unknown mechanism. Murray *et al.* [60] observed that the level of cathinone is related to a sense of satiety, but it does not affect the level of the two hormones which regulate feelings of hunger and satiety, ghrelin and Peptide YY. Girma *et al.* have conducted a study on 385 people, fat mass index (FMI) and body mass index (BMI) resulted lower in chewers than in non-chewers (186 people) [61]. Khat also causes effects including, increased blood pressure associated with a rise in the occurrence of vasospasms, acute myocardial infarction, unfavorable cardiovascular effects and it can also determine problems related to the gastrointestinal tract: esophagitis, gastritis, a delay in intestinal absorption and the development of lesions in the white keratotic oral site of chewing [56,62]. In the Pateria *et al.* [63] review, many cases of acute liver failure (ALF) have been described. In some studies [64,65,66], many cases of autoimmune hepatitis among consumers of *Catha edulis* have also been found. However, causality assessment through the use of RUCAM (CIOMS scale) has not been systematically applied to the suggested cases. There are only a few articles that show the interaction of khat with diseases and respective therapies in Africa. In southwest Ethiopia, many people with HIV are also khat chewers. Soboca *et al.* [67], have found in these patients, a lower response to medicines and to antiretroviral therapy (ART). Ketema *et al.* [68] have studied anti-plasmodial activities on Plasmodium berghei ANKA (PbA), in the malaria infection on Swiss albino mice, orally treated with crude khat (*Catha edulis*). Khat proved to be a poor anti-plasmodial, suppressing only 10% of the parasites without lessening the main symptoms of the disease. Another study of Ketema *et al.* [69], aimed to evaluate the chewing of khat, the onset of severe malaria syndromes, and the immune response during the disease in areas where malaria and this drug, coexist. The IgM and IgG antibody titers and the incidence of jaundice and kidney failure were higher among the consumers, compared to non chewers.

### 1.4. Aims

The main aim of this paper is to focus on the diverse possible pattern mechanisms of hepatotoxicity induced by kava, kratom and khat (“the 3Ks”), while trying to clarify the numerous aspects that still need to be addressed. For this purpose, the Authors will also provide updated available information regarding the constituents of each of the three herbals.

## 2. Results

### 2.1. Constituents

#### 2.1.1. Kava

**Figure 4:**
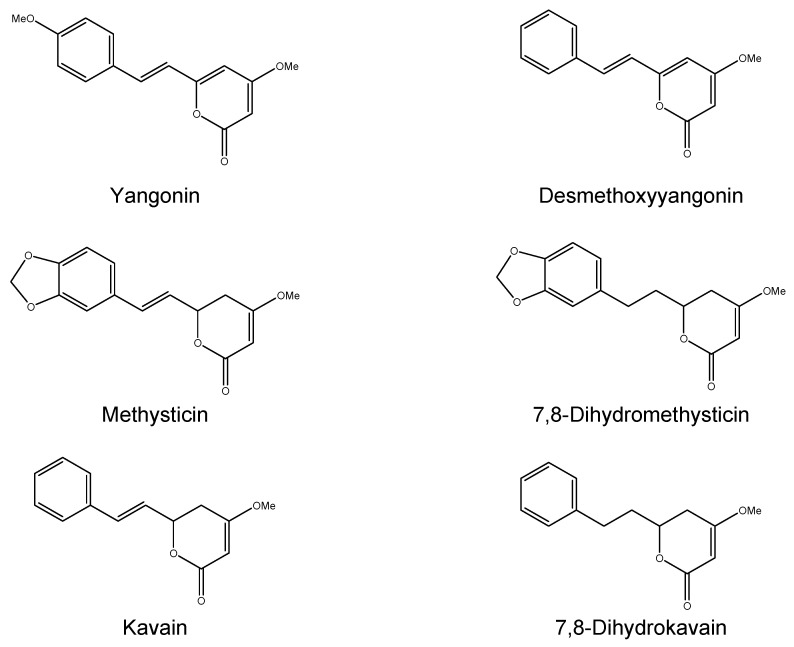
Chemical structures of kavalactones: yangonin, desmethoxyyangonin (DMY), methysticin (M), 7,8-dihydromethysticin (DHM), and kavain, 7,8-dihydrokavain.

**Figure 5:**
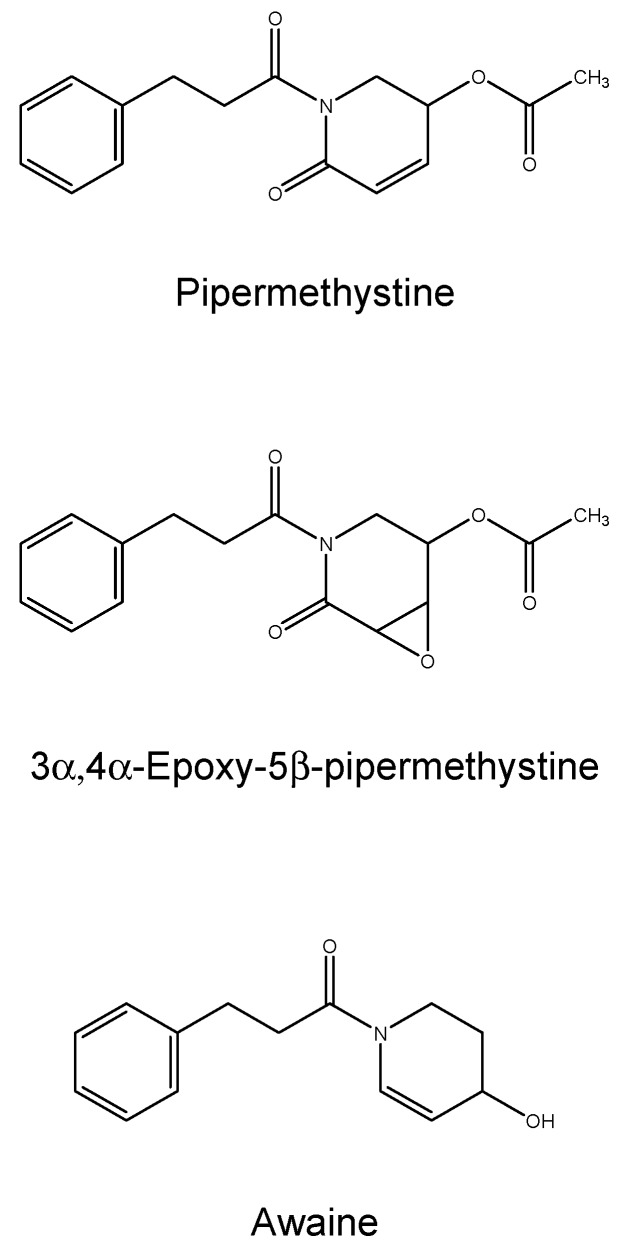
Chemical structures of pipermethystine (PM), 3α,4α-epoxy-5β-pipermethystine and awaine.

**Figure 6:**
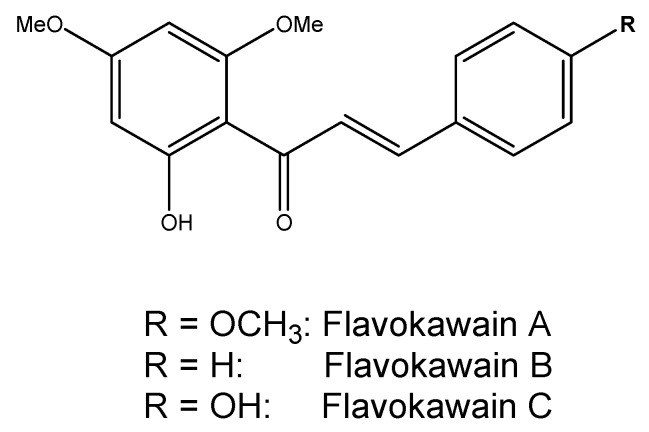
Chemical structures of flavokawains (FK) A, B, and C.

The extraction of kava frees several molecules, the roots are rich in kavalactones (at least 18 different kinds) which determine pharmacological activities (sedation, intoxication, *etc.*) [12]. Nearly all of the psychotropic effects can be attributed to the six major kavalactones, which interact with γ-Amino Butyric Acid (GABA) activity, inhibit monoamine oxidase B and the dopamine and noradrenaline reuptake in the central nervous system (CNS) [70]. Kavalactones dissolve slowly in water and are more rapidly extracted and concentrated in organic solvents; therefore, commercial products (extracted from the latter) usually result in higher concentrations of kavalactones (in comparison with the aqueous solutions) ranging from 30% up to 70% and are 30 times higher than the traditional aqueous extract [71]. In the production of the concentrates, two major techniques are predominant: when kava is dissolved in an ethanol/water combination, the resultant extract contains nearly 30% kavalactones, whereas alternatively when kava is extracted via an acetone–water mix, there is a mean kavalactone concentration of about 70% [72]. Deviations from these extraction procedures and variations among the kava raw materials could impact the relative ratios of kavalactones. Products containing synthetic racemic kavain are also available on the market [73]. Major kavalactones structures, including yangonin, desmethoxyyangonin (DMY), methysticin (M), 7,8-dihydromethysticin (DHM), kavain, and 7,8-dihydrokavain [12], are shown in Figure 4. They represent 96% of kavalactones found in kava roots [11]. Moreover, at least three alkaloids have been characterized in the aerial part of the plant: awaine, pipermethystine (PM) and 3α,4α-epoxy-5β-pipermethystine (shown in Figure 5). Only one cultivar out of the 11 tested (*P. methysticum* cv. Isa) contained the latter compound, and this kava plant variety is seldom used to prepare the ceremonial drink because it causes protracted nausea [74]. Several differences have been observed in the amount of constituents and distribution in the different varieties of the *Piper methysticum* plant. The chemical compositions “Chemotype” can be codified by listing in a diminishing order of proportion the major kavalactones found in the extract, chemotype is genetically determined and depends mainly on the cultivar, and the quality is predominately due to the kavalactone concentration and chemotype [75]. Over 200 different strains of kava have been recognized by chemo-typing, but the chemical signature can vary among the plant portions (rhizomes, roots, basal stems) [76]. This great variability, along with the lack of standard protocols for the preparation of kava extracts, leads to inconsistent evidence of toxicity. The plant also contains minor constituents in the amount of <1% of the dry weight, called flavokawains (FK) A, B, and C (chalcones are shown in Figure 6).

#### 2.1.2. Kratom

**Figure 7:**
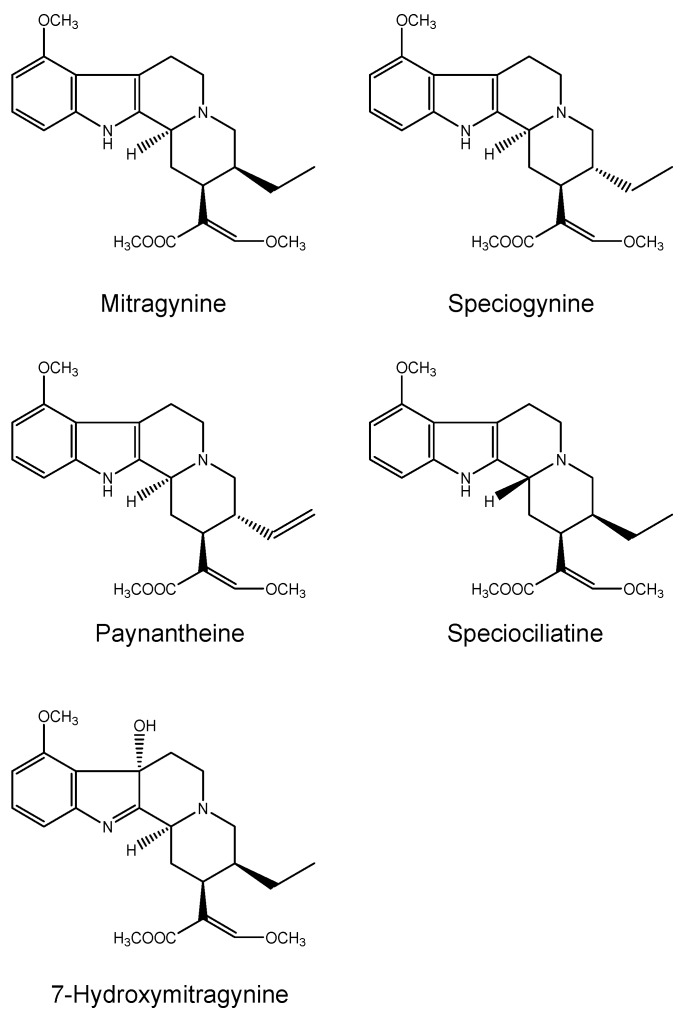
Chemical structures of the most abundant indole alkaloids in *M. speciosa*.

Since the 1960s, more than 25 alkaloids [77] have been isolated and characterized from *Mitragyna speciosa* [32]: the alkaloid content is approximately from 0.5% to 1.5% [37] and it varies from plant to plant [52] depending on the age of the plant tissues, the seasonality and the geographical location. Mitragynine is the most abundant active compound, representing approximately 66.2% [78] of the crude base total alkaloid content [52], that also consists of other minor indole compounds structurally related to yohimbine and *Uncaria* alkaloids [32,37,42,79]. Mitragynine was first isolated in 1907 by D. Hooper [80] and its structure was fully determined in 1965 by X-ray crystallography by Zacharias [81]. It was initially considered to be the prime cause of the antinociceptive kratom effects, since it has opioid-like effects 13-fold stronger than morphine, even though its chemical structure is dissimilar to opiates. Several studies [79,82,83] have demonstrated that another compound, 7-hydroxymitragynine, which represents only a small percentage of the total alkaloid content of the plant extract, presents analgesic effects on the central nervous system 40 times more potent than those of mitragynine [37,78,79,84], probably due to its lipophilicity, which facilitates the crossing of the blood–brain barrier [85]. The second most abundant alkaloid is paynantheine (about 10%), followed by speciociliatine and speciogynine, isolated in percentages lower than 9%. 7-HMG represents only approximately 2% of the total alkaloid content and other compounds (mitraphylline, rhynchophylline, mitralactonal, raubasine and mitragynaline) [37] were observed in a percentage lower than 1%. The most representative alkaloid agents found in kratom extract are reported in Figure 7.

Another alkaloid described and determined with spectroscopic analysis and chemical conversion by Kitajima *et al.* in the fruits of the *M. speciosa*, is 7-hydroxyspeciociliatine [86]. For the total alkaloid profile of *M. speciosa Korth.*, see Hassan *et al.* [32]. The stimulating and analgesic/depressive effects, caused by kratom consumption are mainly due to the presence of these numerous alkaloids: the modulation of the above-mentioned effects is closely related not only to the dosage of consumption but also to the strain of the leaves, since the alkaloid content varies also in relationship to the veining varieties. In nature, there are three different leave strains: the red variety, originating from Bali, is very efficient in pain relief, while the white one and green ones, originating from Malaysia, cause the onset of strong stimulant effects. Other varieties of kratom are sold on the internet like Bali kratom, Malaysian kratom, red, green or white vein Thai kratom, Maeng Da kratom, white-veined Borneo kratom, New Guinea kratom, Java kratom, Sumatra red, the Rifat strain, the bumblebee strain, and red and green Riau [52]. Furthermore, for each of these types, there are several grades of potency: the “organic commercial grade” (the least potent), the “premium” or the “instand instant”, the “super” and the “super enhanced” [87]. Little is known about the real potency of these preparations, except for the experiences reported by the users in the web fora.

The ability of mitragynine to cause analgesic effects is linked to its interaction with supraspinal μ- and δ-receptors [32,37,42,84], while the onset of stimulant activity is due to the block of the stimulation of serotonergic 5-HT_2A_ receptors and to the postsynaptic stimulation of the α-2 adrenergic receptors [84,88]. The antinociceptive mechanism of the 7-hydroxymitragynine is, instead, linked to its capacity of interaction with the μ-receptors, as shown by studies carried out on the tail-flick tests on mice, and partially by the affinity with κ-opioid receptors [89]. Kong *et al.* [90] evaluated the effect of the *M. speciosa* extract (MSE) on human recombinant cytochrome P450 enzymes: he observed that MSE shows a high inhibitory effect on CYP3A4 and CYP2D6, whereas a moderate inhibition was found for CYP1A2 and a weak inhibition for CYP2C19. These results suggest that the use of kratom could cause dangerous consequences if consumed concomitantly with other drugs that are substrates of the same enzymes. Many adverse effects are reported in the literature by several authors, including fatal cases caused by co-consumption with other substances, like carisoprodol [88], modafinil [91], propylhexedrine [92], *Datura stramonium* [93], fentanyl [88], diphenhydramine, temazepam [94], caffeine, morphine and *O*-desmethyltramadol [37,49,88].

However, there are few studies in the literature proving the toxicity of kratom in humans. Seizure episodes and withdrawal syndromes reported are mostly related to chronic consumption of kratom or to acute overdose cases [37,42,54]. The hepatotoxicity reports [54,95] are also very rare and mostly linked to long-term kratom consumption or to its excessive intake.

#### 2.1.3. Khat

**Figure 8:**
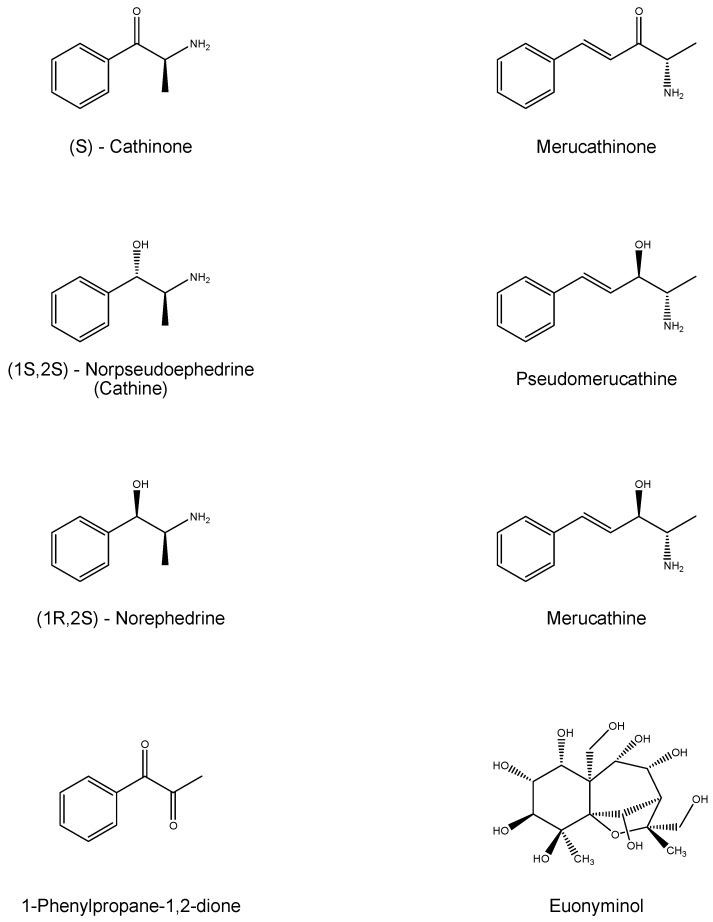
Chemical structures of the most abundant alkaloids present in leaves of *Catha edulis.*

**Figure 9:**
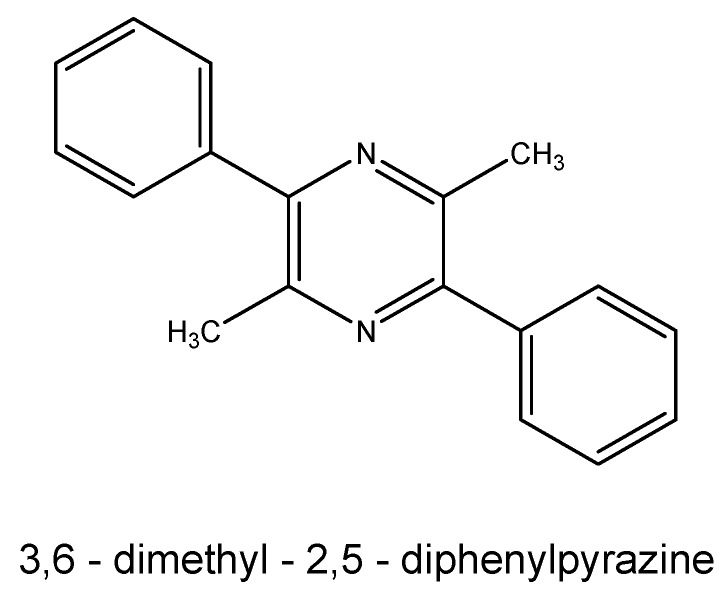
A dimer that is formed as a result of the decomposition of cathinone.

In the khat plant, many different substances have been found: alkaloyds, terpenoids, flavonoids, steroids, glycosids, tannins, amino acids, vitamins and minerals. The climatic conditions and environment determine a different chemical profile of the khat leaves. The major classes of alkaloids in the leaves are: cathedulins and phenylalkylamines. Cathedulins are based on a euonyminol (1,2,3,4,6,8,9,13,14-nonahydroxydihydro-b-agarofuran) structure, which is esterified with acids. In the leaves of khat, 62 species of different cathedulins were identified [96,97]. The most important phenylalkylamines found in the leaves are (*S*)-cathinone, two diastereoisomers, (1*S*,2*S*)-norpseudoephedrine (catina), (1*R*,2*S*)-norephedrine, and 1-phenyl-1,2-propanedione (probably precursor of (*S*)-cathinone) [97,98,99,100]. The biosynthetic process is not well characterized in plants but probably, the synthesis of (*S*)-cathinone starts with l-phenylalanine amino acid. Cathine and (1*R*,2*S*)-norephedrine are obtained by means of the subsequent oxidation process of (*S*)-cathinone [98,99]. This oxidation is obtained during ripening, because cathinone enters the young leaves and its sprouts [99]. The foliage contains cathina and (1*R*,2*S*)-norephedrine approximately in a ratio of 4:1 [101], other phenylalkylamine, merucathinone, pseudomerucathine and merucathine, are present but they are secondary alkaloids with lower stimulant effects. Only in 1975 did the United Nations laboratories identify (*S*)-cathinone as a principal constituent of khat, with a euphoric and anorexic property [99,102]. Cathinone has a tendency to decompose shortly after harvest, forming its dimer, 3,6-dimethyl-2,5-diphenylpyrazine, for this reason, consumers prefer to chew fresh leaves. Figure 8 shows the chemical structures of the most abundant alkaloids present in leaves of *Catha edulis*, whereas Figure 9 shows a dimer that is formed as a result of the decomposition of cathinone.

### 2.2. Hepatotoxicity

#### 2.2.1. Kava

##### Human Studies on Kava Intake

Clough *et al.* [16] carried out a study in the aboriginal population of the east Arnhem Land (northern Australia). One hundred and one adults were recruited in the project, among which 38 (current kava users, four women and 34 men) reported to have used kava at least once during the month preceding the interview. Nine women and 18 men, 27 in all, declared recent use and 36 (23 women and 13 men) reported to have never used kava. Among the kava users, the authors noticed the following significant alterations: a characteristic dermopathy, elevation of γ-glutamyl-transferase (γ-GT) and alkaline phosphatase levels and a decrease in lymphocytes. Russmann *et al.* [103] described the onset of two hepatitis cases with a marked elevation of transaminases and bilirubin after the intake of traditional kava extracts. The dosage of kavalactones was known only in one patient: 18 g/week for 4–5 weeks. After observing that in 27 heavy kava users in New Caledonia there was an elevation of γ-GT in 23 out of 27 and a minimal elevation of transaminases in eight out of 27, the authors concluded that the traditional aqueous kava extracts could also be responsible for liver damage in rare cases. The raise in γ-GT has been frequently described in literature in association with kava drinking [104]. However, the increase was found to be dose related and reversible after kava withdrawal. The γ-GT elevation was observed by other authors [105] at an average ingestion of 310–440 g/week of kava powder and Mathews *et al.* [15], found it to be greatly increased in very heavy kava users (mean consumption, 440 g/week), while Brown *et al.* [106] found γ-GT to be significantly high in chronic kava intake in 65% of cases. However, the γ-GT elevation could also reflect enzymatic induction, rather than hepatotoxicity. The clinical trials performed to examine the efficacy of kava did not show hepatotoxicity. A meta-analysis by Pittler *et al.* [107] including seven clinical trials did not indicate the occurrence of toxicity in the liver. Sarris *et al.* [108] performed a clinical trial on 60 participants who had been suffering from generalized anxiety for a month or more, to assess the efficacy of an aqueous kava product. The dose administered was of 250 mg of kavalactones per day for three weeks. No serious adverse reactions were noticed, no clinical hepatotoxicity was observed in the study. The same author [109] performed another controlled trial, administering 240 mg/kavalactones per day to the patients (affected by generalized anxiety disorder) for six weeks, *vs.* a placebo group. No relevant changes across groups were noticed in the liver function tests.

##### Toxicity Studies

Nerurkar *et al.* [110] evaluated the toxicity of PM *in vitro*, on human hepatoma (HepG2) cells, comparing them to the toxicity of kavalactones. The fact that traditionally only roots are used to prepare the kava drink and as they have no hepatotoxic effects, it allows the authors [74] to suppose that the alkaloids contained in the leaves may be the underlying reason that causes the hepatotoxicity of the kava dietary supplements, as these may contain leaf material. They tested the following alkaloid: PM showing that the exposure of HepG2 cells to 100 μM of PM determined 90% cell vitality loss in 24 h, while an exposure to 50 μM caused a 65% viability loss in the cells. Similar amounts of the following kavalactones, DMY and DHM, did not distress cell viability, even if administered for eight days. Moreover, the authors noticed that PM was able to decrease ATP levels in cells, to reduce mitochondrial membrane potential and to induce apoptosis (release of caspase-3). However, PM was not detected in a series of products containing kava in the German market [70,111,112]. Other authors [113] evaluated the hepatotoxicity of kavain, M and yangonin *in vitro* on HepG2 cells. Cytotoxicity was established by the use of the following assays: lactate dehydrogenase (LDH) and ethidium bromide (EB). Moreover, cell death pattern was evaluated in fluorescence microscopy, and an *ortho*-phthalaldehyde (OPT) fluorescence assay was used to measure glutathione (GSH) oxidation. Among the three compounds, kavain was found to be slightly cytotoxic, while M displayed moderate toxicity depending on the dose (cell vitality decreased to almost 75% at the concentration of 200 μM). Yangonin showed the most marked effects determining 40% of reduction in cell vitality at the concentration of 25 μM. After evaluation with fluorescence microscopy, it was found that the predominant death model was apoptosis rather than necrosis. Zhou *et al.* [114] examined the toxicity of several kava constituents on HepG2 cells. Experiments, carried out exposing cells to the major kavalactones did not show toxicity at a concentration up to 150 μM, excepting for yangonin, which exhibited a weak cytotoxic effect. On the contrary, the three chalcones (FKA, B and C) displayed important cell death at a concentration ranging from 10 to 50 μM. Flavokawain B (FKB) was found to be the most powerful toxin. All the compounds were also tested on a non-tumor origin immortalized human liver cell line (L-02) and only FKB and flavokawain C (FKC) were able to determine cell death in this specific cell type. Therefore the authors demonstrated that FKB is a strong hepatocellular toxin, its mechanism of action is displayed through oxidative stress induction, GSH depletion, by the inhibition of NF-κB transcription *in vitro* and *in vivo* (its activity is crucial in protecting liver cells during their development and survival). Moreover, it causes the constitutive TNFα-independent stimulation of protein kinase (MAPK) signaling pathways (ERK, JNK and p38). The transitory activation of the latter causes proliferation, whereas prolonged activation of JNK is linked to hepatocytic death. The administration of exogenous GSH rescued hepatocytes from FKB induced death. Lüde *et al.* [115] studied the effects of three kava preparations, an acetonic and methanolic root extract and a leaf extract in methanol, on rat liver mitochondria and HepG2 cells. Each preparation showed cytotoxicity starting at a concentration of 50 μg/mL for the lactate dehydrogenase leakage test and of 1 μg/mL for the 3-(4,5-dimethylthiazol-2-yl)-2,5-diphenyltetrazolium bromide (MTT) test. Apoptosis was induced by the three extracts at the concentration of 150 μg/mL. The results showed that kava extracts are toxic for the mitochondria, inhibiting the respiratory chain, decreasing mitochondrial membrane potential and increasing reactive oxygen species (ROS) production. Jhoo *et al.* [116] evaluated the cytotoxicity based on LDH, MTT, and aspartate aminotransferase (AST) enzyme leakage assay techniques on HepG2 cells, of kava (root, stem peeling and leaves) extracts (with methanol). The residues underwent partition with solvents of different polarities “hexane, ethyl acetate, *n*-butanol, and water”. The fraction exhibiting the highest cytotoxic effects was the hexane fraction of the root. Fractions extracted with organic solvents displayed greater toxicity in comparison with the water extracts. Further analysis on the hexane fraction revealed that the molecule responsible for cytotoxicity was FKB. Other authors [117] investigated the effects of the latter molecule on leiomyosarcoma (LMS) cell growth, to examine its effectiveness in the management of uterine LMS. Three different cell lines were used: endometrial adenocarcinoma (ECC-1), uterine leiomyosarcoma (SK-LMS-1), and human endometrium fibroblast-like (T-HESC) (non-malignant). The cells were exposed to different concentrations of FKB, and subsequently apoptosis, cell cycle and viability were evaluated. FKB selectively inhibited the growth and induced apoptosis in ECC-1 and SK-LMS-1 cell lines in comparison with T-HESC control cells. FKB was able to arrest the cell cycle and to induce apoptosis in ECC-1 and SK-LMS-1 cell lines, for these reasons it is suggested to be a potential therapeutic instrument for the handling of uterine LMS. Other authors [118] investigated the toxicity of kava, alone or in combination with acetaminophen (APAP) in an animal study on mice. The administration of kava alone did not cause adverse effects, even if it was administered at the dosage of 500 mg/kg, for a long period of time (six days a week × 14 weeks), while pretreatment with kava for three days, heightened APAP-induced hepatotoxicity, resulting in alanine aminotransferase (ALT) and AST elevation in serum and an enhanced severity of lesions in the liver. The authors also investigated the effects of the chalcones FKA and FKB and DHM (kavalactones group). The two chalcones demonstrated a synergism of action with APAP in the damaging of the liver, while DHM had no such outcome. Therefore, the authors hypothesized that flavokawains played a central role in liver damage, highlighting moreover, that usually these compounds are not abundant in the traditionally utilized cultivars of kava unlike the cultivars that are not recommended for traditional use. Moreover, an analysis performed by the same authors on a set of kava products showed that the amount of FKA and FKB is characterized by a great variability (∼20-fold) [119]. Additionally they evaluated the variability in chemical composition of the extract and the cytotoxicity of 25 kava products, performing a study on human lung adenocarcinoma cells (A549). The results revealed great variability on both the chemical composition and toxic effects on cells. Other authors [120] assessed the presence and the amount of FK (A, B and C) in different cultivars of kava (*wichmannii*-wild, medicinal, noble and “two-days”) concluding that the ratio between FKB and kavalactones is higher in two-days than in the *wichmannii* cultivars of kava, 0.39 and 0.32, respectively, while noble and medicinal cultivars showed a ratio of 0.09 and 0.10, respectively. The kava plant chalcone, FKA and other kava constituents have been investigated in several toxicity studies as chemotherapeutic agents because in countries where the use of traditional kava use is widespread, the incidence of cancer is lower than in other countries. Therefore Li. and colleagues [121] performed a systematic determination of FKA safety on mice. The animal diet was supplemented with 0.6% of FKA or 0.6% kava root extract (KRE) for three weeks. After histopathological investigation of the organs (thymus, kidney, liver, colon, lung, spleen and heart), no signs of toxicity due to FKA were noticed, but there was a KRE diet supplement induced nodular liver proliferation. Other authors [111] did not notice liver injury or any enhancement of galactosamine-induced hepatitis after administering kava (aqueous and organic solvents extracts) to rats at the following doses: 31.25, 62.5 and 133 mg/kg diet for three months. This result is in accordance with a former study of Singh and Devkota [122] performed using the aqueous kava extract. Moreover, Sorrentino and colleagues [26] did not notice liver toxicity after the administration of 7.3 or 73 mg/kg of kava extract (ethanolic) to rats for three and six months. The administration for 14 weeks to rats via gavage of kava extracts (at the following concentrations: 0, 0.125, 0.25, 0.5, 1.0, and 2.0 g/kg/day, five days a week) performed by the National Toxicology Program (NTP) 2007 [123] showed the following results: a raise in γ-GT was noticed in the rats in which the extracts were administrated with a concentration of 2.0 g/kg (males) and from 1.0 up to 2.0 g/kg (females); elevation in serum cholesterol levels in both males and females starting from the dose of 0.5 g/kg; heightened frequency and severity of hepatocellular hypertrophy (HP); and increased liver weight (starting from the dosage of 1.0 g/kg for males and 0.5 g/kg for females). Therefore, the authors highlighted that the value of 0.25 g/kg is the level in which no adverse effects are observed. However, it was also noticed that the incidence ratio of kava-associated hepatotoxicity in humans is really low (0.008/1,000,000 daily doses), whereas the frequency of benzodiazepine-related hepatotoxicity is much higher (bromazepam 0.90, oxazepam 1.23 and diazepam 2.12) [124,125].

##### Carcinogenicity, Mutagenicity and Toxicity

Whittaker *et al.* [126] evaluated the mutagenicity and toxicity of the two commercial products of kava together with the six major kavalactones on mouse lymphoma cells (L5178Y) with pooled human liver S9 and no mutagenic reaction was observed. The most toxic results were obtained with yangonin and DMY at the following doses, 0.5–20 μg/mL and 1.25–20 μg/mL, respectively. Behl *et al.* [127] studied the effects of kava administration in rats and mice via gavage for two weeks, 13 weeks and two years. It was shown that the administration of kava at 0.125 to 2 g/kg in pre-chronic studies could determine an increase in liver weight according to the dose, and an elevation in the frequency of hepatocellular hypertrophy (HH), while in the chronic management, the incidence of HH increased up to 1 g/kg body weight, moreover centrilobular fatty change accompanied HH. No increase in carcinogenesis was observed in the livers of rats, while in male mice an elevation in the frequency of hepatoblastomas (dose-related) was noticed. In female mice, hepatocellular carcinoma and adenoma were increased, however not in the high dose group but in the mid and low dose groups. Moreover, numerous non-neoplastic lesions were found in the livers. Another study performed in 2012 [128] confirmed a remarkable increase in liver cancer in mice of both sexes and the occurrence of non-malignant lesions in the liver, such as HH. Moreover, kava extracts may determine an increase in testicular tumors in rats.

##### Glutathione (GSH) Depletion, Enzymatic Polymorphisms and Cyclooxygenase-Inhibition

Yang *et al.* [129] co-treated rat primary hepatocytes with kava and Acetaminophen (APAP) the co-administration determined 100% loss in cell vitality while treatment with the substances alone determined 50% of loss with APAP and 30% with kava. The reduction in GSH due to APAP was furthermore potentiated by kava. Cellular ATP content and the potential of mitochondrial membrane decreased after the co-exposure. The production or reactive oxygen species (ROS) was found to be higher. Li *et al.* [8] investigated hepatotoxicity mechanisms underlying kava and alcohol intake, as the two substances are frequently co-ingested. The authors suggest a paracetamol-like mechanism of hepatotoxicity for kavalactones, as both molecules are able to determine GSH depletion, moreover they cause CYP2E1 induction, up to 3–4-fold [130], during chronic alcohol intake. The consequent metabolism through this cytochrome, could cause the formation of reactive compounds. The latter could additionally mediate immune toxicity via the production of protein adducts, which function as neo-antigens leading to idiosyncratic responses. Moreover, as suggested by Li [131], several xenobiotics, able to determine immune-related hepatotoxicity, undergo activation through CYP 2C9, 2E1, and 3A. The authors [8] conclude that chronic alcohol intake together with the ingestion of kavalactones, may speed up the production of reactive metabolites with a consequent higher risk of hepatotoxicity. Whitton *et al.* [71], studied the effects of kavalactones alone or in combination with GSH, incubating at 34 °C amoeba cells for seven days. The 100% of the cells incubated only with kavalactones (100 mg·mL^−1^) died, while the 60% of amoeba cells incubated also with GSH, survived after the treatment. Therefore, GSH provides protection against the damages due to kavalactones, via Michael reaction, which results in the opening of the lactone ring rendering it non-toxic for eukaryotic cells. Usually, these substances would therefore be detoxified through the conjugation of GSH to the lactone and across CYP2D6 (main metabolic pathways for kavalactones are demethylation of the alpha-pyrone ring system (4-methoxyl group) and/or reduction (of 3,4-double bond) [132], for this reason individuals with a deficit in CYP2D6 could suffer from liver damage after kava ingestion [133].

However, liver injuries could be prevented by the intake of an excess of GSH. The authors conclude that in all traditional kava preparations the availability of GSH (ratio 1:1 with kavalactones) counterbalances the presence of kavalactones while western extracts are relatively deprived of GSH. Moreover CYP2D6 poor metabolizers, undergoing kava-related hepatotoxicity were described by Russmann *et al.* [134]. Interestingly only 1% of Pacific/Asian islanders are poor metabolizers of this Cytochrome, compared to 12%–21% of Caucasians [135]. This fact could explain the lower incidence of kava-related hepatotoxicity in the Pacific islands. Aghdassi *et al.* [136] identified four patients who underwent acute liver failure after the intake of kava-kava, administered for mild depression. Three of the patients needed liver transplantation and only one of them had previously taken other medications (piretanide and etilefrine). As the polymorphism of the gene CYP2D6 (poor metabolizer) has been related to a higher probability of developing kava-induced hepatotoxicity [134] and, moreover, herbal products such as kava, can determine an inhibition in the catalytic activity of CYP2C19, the authors have investigated uridine diphosphate glucuronosyltransferase (UGT1A7) gene variations. The authors hypothesized that as kavalactones are excreted via the urine for more than 50%, glucuronidation may be a major elimination pathway for *Piper methysticum* metabolites. The authors concluded that the allele frequency of the uridine diphosphate glucuronosyltransferase isoform UGT1A7*3 (characterized by low enzymatic activity) was higher in the patients, compared to the allele frequency in Caucasians; moreover, one of the patients needing transplant was found to be homozygous. No CYP2D6 decrease in activity was assessed because no poor metabolizer phenotype was detected for this enzyme. In reply [27] to the previously reported study, Stickel highlighted that caution is needed when claiming an association, having analyzed only a limited case series of four patients. Also cyclooxygenase-1 and 2 (COX-1 and COX-2) inhibition could be responsible for kava hepatotoxicity [137], since yangonin and dihydrokavain inhibit these enzymes at the concentration of 100 μg/mL. Another study [138] demonstrated that kava inhibits COX-2 selectively. These effects would commonly be associated with benefits and antioxidant properties, however according to other authors, hepatotoxicity is not an unusual finding after exposure to drugs that inhibit COX [72].

##### P450 Activity Alteration

Mathews *et al.* [139] evaluated the inhibition potential of the whole kava extract (100 μM total kavalactones) on human liver microsomes (HLMs). A decrease in P450 activities associated with the concentration was noticed. A substantial inhibition in the activity of the following CYP was detected: CYP2C9 (92%), 2C19 (86%), 1A2 (56% inhibition), 2D6 (73%), 3A4 (78%), 4A9/11 (65%) after the pre-incubation with HLMs and NADPH for 15 min. The incubation with kawain did not alter enzymatic activity while DMY inhibited CYP2C9 substantially (42%), M (58%), and DHM (69%) at 10 μM. Moreover, the latter molecules could inhibit CYP2C19 (DHM 76%), CYP2D6 (M 44%) and CYP3A4 (DMY 40%, M 27%, and DHM 54%). These findings show that kava is extremely likely to determine drug interactions through its inhibition of CYP450 enzymes. Another *in vitro* experiment [140] performed on cryopreserved human hepatocytes and on cDNA-expressed human enzymes, revealed that the kava extract is a powerful inhibitor of CYP 1A2, 2C9, 2C19, 2E1, and 3A4. Moreover, the compounds displayed moderate cytotoxicity on human hepatocytes. M, exhibited more potent cytotoxic effect and inhibition activity followed by kava root extract, DMY, and yangonin. Different extracts of kava were likened to evaluate their inhibition potency on major drug metabolizing P450 enzymes on human liver supersomes and microsomes by Côté *et al.* in 2004 [141]. Inhibition in activity was noticed for CYP3A4, 1A2, 2C9, and 2C19. The inhibition was more marked in the commercial preparation while the aqueous extract was found to be less potent. Mathews *et al.* [142] performed experiments *in vivo* and *in vitro* to evaluate the effects on CYP450 and on *P*-glycoprotein activity due to kava extracts and kavalactones. Oral pharmacokinetics of kawain were also assessed in rats. Kawain underwent extensive absorption and was eliminated (>90% of the dose) within 72 h mainly in urine. A seven-day pre-treatment with kava (391 mg/kg) induced a significant increase (35%) in Hepatic P450 content. The activities of the following cytochromes, CYP1A2, 2B1, and 2C6, were heightened about 200% in comparison with control animals. An increase (51%–80%) in the activity of CYP3A1/2 was also noticed, while the activities of CYP2D1 (human CYP2D6 homologue) and CYP2C11 were reduced (25% and 77%, respectively). *In vitro* experiments on human hepatic microsomes showed inhibition of CYP2C9, CYP2C19, CYP2D6 and CYP3A4. There was a modest stimulation of *P*-glycoprotein activity. Another study performed on rats [143] was carried out administering PM 10 mg/kg and kava root/rhizome extracts (KRE) 100 mg/kg (acetone/water extracts) for fifteen days. This treatment did not cause any substantial alterations in liver weight, liver function tests, nor any important hepatic toxicity was noticed through measuring anti-apoptotic protein Bcl-2, Bax pro-apoptotic protein and malondialdehyde (MDA) as apoptosis and lipid peroxidation markers. Nevertheless, rats treated with PM showed changes suggesting adaptive response to oxidative stress (rise in TNFα mRNA expression, hepatic GSH, cytosolic superoxide dismutase-Cu/ZnSOD and increase in CYP1A2, CYP2E1). Guo *et al.* [144] studied the effects of kava administration via gavage for 14 weeks in male rats to evaluate the changes in the expression of gene encoding for drug metabolism enzymes. In the high-dosage treatment groups 16 genes were found to be altered. CYP3A1, 3A3, 1A1, 1A2, and 2C6 expression increased, while CYP 2C40 and 2C23 decreased in rat livers. The changes in expression were found to be dependent on the dose. Yamazaki *et al.* [145] investigated the effects on the gene expression of CYP1A hepatic isoforms in rats, following the administration of kava products for eight days (380 mg/kg/day kavalactones). CYP1A is probably involved in the activation of carcinogens such as aflatoxin and benzo(a)pyrene. After the treatment, liver weight increased significantly. A moderate rise in CYP1A2 mRNA expression was observed, while CYP1A1 mRNA expression was found to be markedly enhanced (75–220-fold). Guo and colleagues [146] investigated the gene expression profile in male mice livers following the administration via gavage of kava extracts for 14 weeks. An alteration in oxidative stress response Nrf-2 mediated as well as mitochondrial activity were noticed. Additionally, the levels of a substantial number of genes involved in drug metabolism and xenobiotic metabolism pathways were altered. 29 Phase II genes, 38 transporters genes and 28 Phase I metabolizing enzymes genes were found to be significantly changed in their expression after kava intake. Major gene expression alterations were noticed on the following: CYP1a1, CYP1a2, Gstal, Gsta2, CYP2b20, CYP2a5, CYP3a11 and CYP2c55. This fact could lead to potential hepatotoxicity through the interaction between drugs, herbs and modulation of metabolism. After the administration via gavage of kava extracts for 14 weeks to rats, it was shown through immunohistochemical analysis that CYP-450 expression in 2.0 g/kg treated females had decreased for the homolog of CYP2D6 [123]. Moreover, a raise in the expression of CYP1A2, 2B1, 3A1 was noticed in 1.0 and 2.0 g/kg treatment groups (males and females). Alteration of CYP450 activity can determine herb drug interactions leading to toxicity, in fact several hepatotoxicity cases included co-ingestion of other drugs or herbal remedies. Moreover, as the inhibition potential is dissimilar in the different compounds, and furthermore the composition in kavalactones and other constituents is highly variable, depending on extraction procedure and raw material, divergent effects can be expected after the intake of different kava preparations.

##### Reactive Metabolites

Hepatotoxicity could also be mediated through the formation of reactive kavalactones metabolites including 6-phenyl-3-hexen-2-one [147]. It was found to be highly reactive *in vitro*. *In vivo*, the formation of mercapturic acid-conjugates was assessed in the urine of two volunteers after a single dose of powdered kava root (10 g). Moreover, the generation of electrophilic intermediates, such as 11,12-dihydroxykavain-*o*-quinone and 11,12-dihydroxy-7,8-dihydrokavain*o*-quinone, has been demonstrated by Johnson *et al.* [148], after *in vitro* incubation of kava extracts with NADPH, GSH and hepatic microsomes. These products could interact binding to DNA or through alkylation and linkage to hepatic proteins. The mercapturic acids of these species (quinoid) were not detected in human urine after the ingestion of kava. This fact could lead to the conclusion that although the formation *in vitro* of quinoid metabolites has been observed, these compounds are not formed in considerable amounts after the intake of modest doses of kava products. The resultant catechols (dihydroxylated derivatives) were conjugated extensively with sulfate and glucuronic acid and were detected in human urine. The impact of quinoid metabolites could be relevant, and therefore contribute to hepatotoxicity *in vivo*, in case of saturation of conjugation pathways or for the alteration of metabolic routes. Other authors [149], after examining the *in vitro* metabolism of flavokawains, highlight that the chalcone metabolite conjugates could presumably be active *in vivo*, and that currently these metabolites are not included in routine testing.

##### Mould Hepatotoxins and Contaminants

Teschke *et al.* [70] performed a review focusing on PM, flavokavain B and mould hepatotoxins as possibly being responsible for this toxicity. The authors noticed a lack of evidence regarding the two first compounds. The weather that characterizes the Pacific islands (warm and humid) is likely to determine the development of mold (such as aflatoxins or others) during storage. Moreover, kava material may be contaminated by pesticides, fertilizers, oil, bacteria, fungi, *etc*. The authors suggest performing further studies on this topic. This hypothesis has led to a lively debate [150,151].

##### Inflammation

Zhang *et al.* [152] highlighted the presence of hepatic inflammation (as described in literature) in cases of kava intake and kava-associated toxicity. The authors suggest direct inflammation through the action of more than 40 molecules isolated from kava, among which: kavalactones, the toxic alkaloid PM and the chalcone FKB, highly toxic. Otherwise, the inflammation could be due to indirect mechanisms such as the reduction of liver GSH or to the effects of toxic metabolites. Therefore, the same authors published a study in 2012 [153] highlighting the potential engagement of liver macrophages in the development of toxic damage to the liver. Experiments carried out on isolated perfused rat livers, showed that hepatic sinusoids undergoing treatment with kavalactones, displayed extensive injuries, while if pretreated with gadolinium chloride (macrophage intoxicant) no damage was noticed.

##### Kava Hepatotoxicity Reports

Several hepatotoxicity cases are reported in literature. The first cases were described in 1998 [28]. Gow *et al.* [154] described in 2002 the case of a 56-year-old woman admitted to hospital in Melbourne for increasing jaundice that lasted for two weeks and was accompanied by nausea and fatigue. She had been taking an herbal supplement to treat anxiety for three months. The herbal remedy contained 60 mg of kavalactones as well as Scutellaria Laterifloria 100 mg and Passiflora Incarnata 50 mg. Viral tests for hepatitis, cytomegalovirus and Epstein Barr were all negative. Wilson’s disease and paracetamol intake were also excluded. The patient’s condition worsened and she underwent liver transplantation, but after the operation she died of massive bleeding. The explanted liver histological examination revealed considerable hepatic necrosis. Another case was described by Escher *et al.* [155]. A 50-year-old man experienced fatigue, dark urine and darkened skin for about a month, before going to the doctor with jaundice. Although the patient’s medical history was unexceptional and he did not use alcohol or other drugs, he suffered from anxiety, and, for this reason, he took kava extracts (3–4 capsules a day, 210–280 mg of lactones cumulatively) for two months. After undergoing liver transplant, he recovered. Campo and colleagues [156] reported the case of a 14-year-old girl who successfully underwent liver transplantation for fulminant hepatitis after an intake of commercial kava products for three months, while other authors [157] performed a retrospective case series on patients admitted to the liver transplantation unit, describing a high incidence of fulminant hepatic failure among dietary supplements users. They identified three patients who underwent liver transplant after the intake of kava, alone or in association with Ma Huang or Chaparral. Stickel and colleagues [158] analyzed 29 cases of hepatitis following kava intake, reported to the German Department of Pharmacovigilance from 1990 to 2002. Moreover, the authors evaluated seven cases previously described in literature, they concluded that the greater part of the patients were female, hepatic damage (cholestatic hepatitis or hepatic necrosis) occurred equally with both acetonic and alcoholic kava extracts. In addition, the latency of the toxic reaction onset and the cumulative dosage were decidedly variable. Nine patients needed liver transplant because of fulminant hepatic failure, three of them died and the others all recovered after eliminating kava use. The authors hypothesize idiosyncratic and immune-allergic factors to be responsible for kava hepatic damages. Another case is reported by Humberston *et al.* [159] involving a 14-year-old teenager undergoing liver transplant after the use of kava products for four months. Teschke *et al.* [160], highlighted that, if the 19 suspected hepatotoxicity cases supposed to be due to kava intake in Germany had been evaluated more critically, the causality in these cases would have been attributed only to one patient. Possible causality could be endorsed for another patient, unlikely/excluded for five patients and insufficient data for 12. Moreover, the case of a 42-year old male who had toxic hepatitis caused by consuming traditional kava products (2 to 3 L cumulative volume) in the Samoan islands, was reported by Christl *et al.* [161]. Fortunately, the patient recovered.

Up to 2002, 82 cases in total of hepatotoxicity possibly linked to kava are available from different databases. Amongst the cases evaluated by Schmidt [125], 20 had no relationship with kava intake, 21 cases were characterized by concomitant treatment with potential hepatotoxic substances, 31 were characterized by insufficient data and in seven cases there was substantial doubt in considering the causality of kava. Amid these, three were possibly associated to kava. Clouatre [72] suggests that the direct toxicity of kava is small, however the possibility it determines drug interaction or heightens the toxicity of other drugs is huge and kava toxicity seems to be due to idiosyncratic reaction. However, at least three major mechanisms of hepatotoxicity are enumerated in literature: GSH reduction in liver, cyclooxygenase and CYP450 inhibition. Nevertheless, if every kava hepatotoxicity reported case were to be attributed to kava intake, the rate of adverse reactions as calculated by Schmidt [125] may be 0.3 cases/1,000,000 daily doses, therefore kava would display a better risk-to-benefit ratio in comparison with benzodiazepines.

Schmidt *et al.* examined once more kava hepatotoxicity reports up to 2003 [124], as listed by several health authorities among which: EMEA, FDA, BfArM, IKS, Therapeutic Goods Administration (Australia) and from literature. Only three cases could be attributed to kava intake with high probability, two were related to kava overdose. Therefore, kava induced-hepatotoxicity should be really rare, and the authors highlight the fact that, as drug induced hepatotoxicity frequency ranges from 1 to 10 per 100,000 exposed individuals, kava would be under one, even if all the cases of reported toxicity were causally linked to kava intake.

#### 2.2.2. Kratom

##### Toxicity Studies in Cell Lines and in Animal Models

To date, there is scarce information about the toxicity of *M. speciosa* alkaloid extracts and little is known about the adverse effects that have really originated from mitragynine intake. The *M. speciosa* cytotoxicity has been evaluated by three authors in different cell lines [162], brine shrimp [163] and gene mutation assays [164]. Saidin [162] tested the cytotoxicity both of the methanol-chloroform extract (MSE) and of mitragynine (MIT), on human cell lines (HepG2, HEK 293, MCL-5, cHol, and SH-SY5Y). The MSE inhibited cell proliferation in all cell lines, depending on the dose administered, inducing cell death at 1000 μg/mL and MIT showed a similar model of action. Even if the activated pathways that led to cell death were different (MSE cell death seemed not to be associated with p53 and caspases pathway, contrary to that caused by MIT), the results of these studies suggest that both the methanol–chloroform extract and its dominant alkaloid mitragynine, generate cytotoxicity effects at doses higher than 100 μg/mL. In a brine shrimp lethality test, Moklas *et al.* [163], evaluated the toxicity level of three different preparations of mitragynine: the authors found that the LC_50_ for the aqueous extract was 98 μL/mL, the crude alkaloid extract exhibited a LC_50_ value of 62 μL/mL, while mitragynine showed the high toxicity with LC_50_ of 44 μL/mL. The potential of mutagenic and antimutagenic activity of *M. speciosa* was evaluated by Ghazali *et al.* [164], incubating the aqueous extract of the plant with Salmonella typhimurium TA 98 and TA 100 bacterial strains, in presence and absence of the metabolic activator S9 system. The Ames test (Salmonella/microsome mutagenicity assay) showed no mutagenic activities for *M. speciosa* both in presence and in absence of the metabolic activator, and with both bacterial strains, but, in the same experimental conditions, *M. speciosa* had a strong antimutagenic property. Several studies were carried out in animal models to evaluate the toxicity of the mitragynine, Macko *et al.* [165] in 1972, tested, for the first time, mitragynine toxicity in rats and dogs: he found no adverse effects in rats up to a dose of 40 mg/kg/day six days per week, until the twenty-second day of treatment, when hematological alterations were observed, on the other hand, no toxicity signs were noticed in a group of dogs treated with an oral dose of 5 mg/kg/day of mitragynine for three weeks [52]. Recently, Sabetghadam *et al.* [53] investigated the sub-chronic exposure to mitragynine of male and female rats, administering oral doses of 1, 10, or 100 mg/kg for 28 days. At the lower doses, there was no evidence of toxic effects (such as tremors or seizures), whereas at a dose of 100 mg/kg, alterations in food intake emerged, with a consequent strong decrease in weight especially in female rats. No deaths occurred at the maximum dosage. Hematological, biochemical analysis and histopathological examination of the brain, kidneys and liver were performed. With regard to the hematological findings, the authors observed a severe anemia, characterized by a decrease in the red and white blood cells, a reduction of the hematocrit levels with a lowering of the hemoglobin content. Signs of tissue toxicity were observed in the histopathological analysis performed on the brain, kidney and liver. Local vacuolation and the presence of degenerated necrotic neurons were noticed in the brain; in the kidneys an early state of nephrotoxicity was observed. These findings were highlighted in all the animals exposed to the maximum dosage of mitragynine, in particular in female rats. The alteration of some biochemical parameters corresponded to the structural modifications discovered in the liver. Very high levels of serum lactate dehydrogenase, aspartate aminotransferase (AST), alanine aminotransferase (ALT) and urea, indices of hepatocellular damage, were observed; there was also an increase in liver weight of all the animals exposed to the maximum dose of mitragynine. The histological liver examination showed moderate destruction of polygonal lobules, dilation of sinusoids and hemorrhagic hepatocytes; there were no signs of centrilobular necrosis or inflammatory cell infiltration. An increase in triglycerides, cholesterol, AST and ALT values, albumin (indices of hepatic impairment), and the presence of histological evidence for hepatic cellular damages, were also observed by Harizal *et al.* [45] after acute oral administration of 1000 mg/kg of methanolic extract of *M. speciosa* in rats. In all the rats of the treated group, the histological analysis revealed a severe hepatotoxicity, with a major number of Kupffer cells, hemorrhagic hepatocytes, sinusoids congestion, steatosis and centrilobular necrosis. These studies show that the sub-chronic dosages (1–10 mg/kg) of mitragynine in rats, which in humans corresponds to a dose of 0.1 to 1.7 mg/kg, seems to be quite safe when compared to those consumed by kratom users: in fact, the content of kratom juice regularly consumed in the northern regions of the Malaysia Peninsular, is equal to approximately 0.3 to 5.1 mg/kg per day and users do not show any side effects related to the chronic use of this substance, as reported by Vicknasingam *et al.* [41,52].

As mitragynine has proved to be extremely toxic in rats, when administered for a prolonged period at 100 mg/kg, in the future more studies must be carried out on the chronic exposure to mitragynine in more complex living systems with dosages relevant for humans, in order to ascertain the possible link between this substance and the severe hepatotoxicity observed in some of the researches here reported.

##### Kratom Hepatotoxicity Reports in Literature

Literature reports about mitragynine toxicity in humans are rare, even if in recent years clinical cases are increasing. Only two papers have reported cases of hepatotoxicity secondary to kratom consumption. The first case was published by Kapp *et al.* [54] in 2011: they described the case of a 25-year-old man, who after taking kratom for two weeks showed the onset of jaundice and itching. He had started to consume one/two teaspoons of kratom (each teaspoon is approximately 2.3–3.5 g) twice daily, increasing the intake up to four/six teaspoons daily. He interrupted the intake because of swallowing problems, fever and chills and on the fifth day after stopping kratom, he developed severe abdominal pain with the appearance of brown urine, jaundice and itching and was admitted to hospital. The laboratory tests showed elevated values of transaminases, direct bilirubin and alkaline phosphatase: the autoimmune analysis together with the antinuclear antibodies (ANA) test and viral tests for hepatitis were all negative and no further drugs or medications were found. A computed tomography of the abdomen was performed and it showed liver steatosis, without dilation of intra and extrahepatic bile duct, while a liver biopsy revealed the presence of a pure cholestatic injury with bile precipitations and fat vacuoles without hepatocellular damages. Distended and hyperemic sinusoids were observed with signs of inflammation, which led to the diagnosis of canalicular cholestasis. Toxicological analysis were performed in LC-MS with a linear ion trap, on both serum and urine of the patient to detect the main alkaloids of mitragynine and its metabolites: samples of kratom powder found in his home were also analyzed to exclude the presence of contaminants or adulterants. Despite more than two weeks had passed from the kratom discontinuation, as stated by the patient, mitragynine and its main metabolites were detected in the urine sample. Whereas the data available on mitragynine half-life are exclusively related to rats (4–9 h after a single dose) [166,167], the presence of the substance and its metabolites in biological samples (serum and urine) of the patient may be related to a serious prolongation of the alkaloids half-life that could be the consequence of the hepatic injury or to the delayed clearance caused by the extensive first-pass hepatic metabolism. Due to the lack of scientific data on the toxicity of kratom in humans, the physicians could not directly correlate the onset of acute liver disease with the intake of kratom. The effects of the substances contained in *M. speciosa* extract (alkaloids, saponins, flavonoids, *etc.*) have not yet been well researched making the correlation between the health of the liver and the intake of these preparations very difficult: for example only recently Azizi *et al.* [168] demonstrated a correlation between the administration of *M. speciosa* extracts in mice and the increased level of glutathione-*S*-transferase, as a possible sign of hepatic disease. The second case report was described by Dorman *et al.* [95] in 2015: a 58 year old man was admitted to hospital with jaundice and dark urine after prolonged daily kratom intake. He had also consumed other medications, for more than two years, including quetiapine (100 mg/day) and sertraline (50 mg/day). Biochemical analysis revealed a total bilirubin of 25.6 mg/dL, alanine transferase (ALT) 106 U/L, aspartate aminotrasferase (AST) 49 U/L and alkaline phosphatase (ALP) 790 U/L with a R ratio of 0.24 that indicated cholestatic injury. The antinuclear and the smooth muscle antibodies tests were negative as were the viral tests for hepatitis A, B and C. The ultrasound analysis of the abdomen revealed only an irregular hepatic texture without signs of biliary obstruction and a hepatic biopsy was not performed. The authors defined idiosyncratic the onset of liver complication but evaluated as “convincing” its association with the intake of kratom.

#### 2.2.3. Khat

##### Hepatotoxicity

Toennes *et al*. [59], has studied the pharmacokinetics of khat in four subjects and the results suggest that chewing it, is very effective. The buccal mucosa plays a very important role in the absorption of cathinone, cathine and norephedrine. Only 10% was found in the leaves chewed. The amount of norephedrine found in urine was higher than the amount ingested. Only 7% of cathinone was excreted in the urine. In this experiment, a single administration (0.6 g/kg) was given; normally consumers chew 100–300 g of leaves in a period of 3–4 h [59,169,170]. In the United Kingdom, khat is legal and cheap, this is why the use of this substance is very high, especially among Somalis who live in the UK [65]. In the literature, there are an increasing number of cases of severe liver injury as a consequence of khat use or abuse, in particular in the UK, Holland and other European countries where *Catha edulis* is legal. In many cases, a common factor is the occurrence of non-viral hepatitis with khat uses. Roelandt *et al.* describe a case of a 26-year-old Somali man, living in the UK, with secondary acute liver failure. Having excluded all other possible causes such as tumoral invasion, vascular or biliary complications, nodules, cholecystolithiasis, steatosis and hepatitis and the absence of alcohol, medication, dietary supplements, herbs, or illicit drug abuse, apart from khat, a liver transplant was deemed necessary [65]. Six other patients in the UK, with an age ranging from 24 to 57 years, five Somalis and one Yemen, were affected with acute hepatitis. Patients followed a treatment with prednisolone and responded well to immunosuppression. All of these had a history of khat use. In evaluating diverse parameters, such as liver enzymes, autoimmune screen, exclusion of viral hepatitis alcohol and drugs, immunoglobulin levels and liver histology, Riyaz *et al.* [64] suspected that khat could possibly cause the onset of autoimmune hepatitis, in genetically susceptible subjects. It will be necessary to conduct further studies in order to confirm this hypothesis. In the case report of Yildiz *et al.* [66], a 25-year-old male Somali, ex-consumer of khat, went to a medical center with jaundice and hepatitis, which was negative to viral hepatitis A, B and C, but positive to ANA (Antinuclear antibodies) and anti-actina antibodies, even though the biopsy showed a compatibility with toxic hepatitis, it was more similar to autoimmune hepatitis for the serum parameters.

Chapman *et al.* [171] presented six patients from the UK who were khat abusers and had reported severe liver injury, four underwent a liver transplant, and two died. Abid *et al.* investigated whether or not khat induces apoptosis in the L02 human hepatic cell line with the result that khat does indeed induce significant apoptosis of cultured cells L02 [172].

##### Inhibiting Action

The enzymes implicated in metabolism of khat have not yet been described. It is supposed that P450 (CYP) is involved because many recent studies have identified CYP2D6, as the enzyme included in the metabolism of syntethic cathinone derivates [173,174,175]. These substances are both a substrate and an inhibitor for CYP2D6 [176]. As CYP3A4 and CYP2D6 cytochromes are able to metabolize most drugs, the inhibition of these enzymes, could become a significant problem [135]. Bedada *et al.* [177] conducted a comparative study for the evaluation of khat effects on the enzymatic activity of cytochrome P450 (CYP) 2D6 and CYP3A4. The test was conducted on 40 ethiopian volunteers. Dextromethorphane and khat were administered, the first as a probe drug. Genotyping of the CYP2D6*3 and CYP2D6*4 were performed. In conclusion, inhibition of CYP2D6 activity by khat was more important in CYP2D6*1/*1 compared to CYP2D6*1/*4 genotypes. A marginal inhibition of CYP3A4 activity was observed in the presence of khat [177].

## 3. Materials and Methods

The identification of relevant scientific articles was performed via the following research engines: Medline, Cochrane Central, Scopus, Web of Science, Science Direct, EMBASE and Google Scholar, up to December 2015 using the following keywords for Kava: “Kava”, “*Piper methysticum*” “Hepatotoxicity”, “Liver” “Injury”, “Toxicity”. The main keywords “Kava” and “*Piper methysticum*” were individually searched and then again in association to each of the others. The 738 and 773 sources initially found, with “Kava” and “*Piper methysticum*”, respectively, were selected in order to exclude papers not appropriate for the purpose of the review and duplicate sources. Only 74 papers [8,11,12,15,16,26,27,28,70,71,72,73,74,75,76,103,104,105,106,107,108,109,110,111,112,113,114,115,116,117,118,119,120,121,122,123,124,125,126,127,128,129,130,131,132,133,134,135,136,137,138,139,140,141,142,143,144,145,146,147,148,149,150,151,152,153,154,155,156,157,158,159,160,161] (40 research articles, 15 review articles, 2 randomized controlled trials, 9 case report/series, 5 letters to the editor, 1 report, 1 book chapter and 1 meta-analysis) were included in the results.

The following keywords were used for Kratom: “Kratom”, “*Mitragyna speciosa*”, “Mitragynine alkaloids”, “toxicity” and “hepatotoxicity”. The main keywords “Kratom” and “*Mitragyna speciosa*” were individually searched and then again in association to each of the others. The 71 and 115 sources originally found, with “Kratom” and “*Mitragyna speciosa*”, respectively, were selected in order to exclude papers not suitable for the aim of the review or duplicate sources.

Only 33 papers [32,37,41,42,45,49,52,53,54,77,78,79,80,81,82,83,84,85,86,89,90,91,92,93,94,95,162,163,164,165,166,167,168] (5 review articles, 1 letter to editor, 7 case reports, 16 research articles, 1 short communication, 1 Ph.D. thesis, 2 book chapters) were included in the results.

The following keywords were used for Khat: “Khat”, “*Catha edulis*”, “Hepatotoxicity”, “Liver” “Injury”, “Toxicity”. The main keywords “Khat” and “*Catha edulis*” were individually searched and then again in association to each of the others. The 583 and 536 sources primarily found, with “Khat” and “*Catha edulis*”, respectively, were initially identified in order to exclude papers not appropriate for the purpose of this review and duplicate sources. Only 21 papers [59,64,65,66,96,97,98,99,100,101,102,135,169,170,171,172,173,174,175,176,177] (13 research articles, 5 reviews, 3 case reports) were included in the results.

All sources have been screened by three of the authors independently, and in order to be included they had to be selected by at least two of the authors.

## 4. Conclusions

Despite the fact that several experimental studies have already been published in order to show kava mechanisms of hepatotoxicity, including direct toxicity performed by kava constituents, GSH depletion, COX inhibition, reactive metabolites, the interference with CYP-450 enzymes, carcinogenesis studies and the intervention of contaminants, mold hepatotoxins, extraction procedures and inflammation, the mechanisms of kava hepatotoxicity are still not fully elucidated in humans. Moreover, according to the examination of kava toxicity in reports performed by different authors, the occurrence of liver toxicity due to kava products seems to be extremely rare. Its manifestation is suggested to be in relationship with a lack of compliance to recommended dose/overdosing or with a co-ingestion of herbal supplements or other substances or as a consequence of non-standardization of the kava products [178]. Furthermore, the ban imposed in Germany on the herbal supplement kava was overturned in 2014, after the decisions of two administrative German Courts [179,180,181].

Literature reports about mitragynine toxicity in humans are rare, even if in recent years clinical cases are increasing. Only two papers [52,93] reported cases of hepatotoxicity following kratom consumption; the first one was published by Kapp *et al.* [54] in 2011 involving a 25-year-old man, the second one was described by Dorman *et al.* [95] in 2015, involving a 58 year old man.

Although in both cases the available data were significantly suggestive of kratom induced hepatotoxicity, the authors of both studies due to the lack of scientific data on the toxicity of kratom in humans, were cautious in confirming unequivocally this association; in the first case [52] the physicians could not directly correlate the onset of acute liver disease with the intake of kratom, taking into account that the effects of the substances contained in *M. speciosa* extract (alkaloids, saponins, flavonoids, *etc.*) have not yet been fully investigated making the correlation between the state of the liver and the intake of these preparations very difficult, whereas in the second case report [93], the authors defined idiosyncratic the onset of liver complications but evaluated as “convincing” its association with the intake of kratom. However, it still remains to be established if the onset of liver complications could be attributable to kratom alkaloids, extract-production byproducts, or other contaminants [52].

In the literature, there are an increasing number of cases of severe liver injury as a consequence of khat use or abuse, in particular in the UK, Holland and other European countries where *Catha edulis* is legal [62,63,64,169]. In many cases, a common factor is the occurrence of non-viral hepatitis with khat uses. Up to this moment, the enzymes included in metabolism of khat have not yet been fully described, however it is supposed that P450 (CYP) is involved because many recent studies have identified CYP2D6 as the enzyme included in the metabolism of synthetic cathinone derivates [173,174,175].

This review allow us to conclude that if, on the one hand, some of the mechanisms underlying kava hepatotoxicity have been identified, several other aspects still need clarification, while, on the other hand, kratom and khat hepatotoxicity must still be elucidated and only through a careful evaluation of each case together with further experimental studies will it be possible to increase the knowledge in this field.

## 5. Limitations of the Review

A limitation of this review is related to the fact that, although in the literature, the assessment of causality for hepatotoxicity due to kava was carried out through the use of valid assessment methods such as the RUCAM, this evaluation has not been applied systematically in suspected cases of kratom and khat related hepatotoxicity.
